# The role of synovial fluid markers of catabolism and anabolism in osteoarthritis, rheumatoid arthritis and asymptomatic organ donors

**DOI:** 10.1186/ar3293

**Published:** 2011-03-24

**Authors:** Rediet Kokebie, Rohit Aggarwal, Sukhwinderjit Lidder, Arnavaz A Hakimiyan, David C Rueger, Joel A Block, Susan Chubinskaya

**Affiliations:** 1 Section of Rheumatology, Department of Internal Medicine, Rush University Medical Center, 1653 West Congress Parkway, Chicago, IL 60612, USA; 2 Division of Rheumatology, Department of Medicine, University of Pittsburgh, S700 Biomedical Science Tower, 3500 Terrace Street, Pittsburgh, PA 15261, USA; 3 Department of Biochemistry, Rush University Medical Center, 1653 West Congress Parkway, Chicago, IL, 60612 USA; 4 Stryker Biotech, 35 South Street, Hopkinton, MA 01748, USA; 5 Department of Orthopedic Surgery, Rush University Medical Center, 1653 West Congress Parkway, Chicago, IL 60612, USA

## Abstract

**Introduction:**

The purpose of this study was to correlate the level of anabolic and catabolic biomarkers in synovial fluid (SF) from patients with rheumatoid arthritis (RA), patients with osteoarthritis (OA) and asymptomatic organ donors.

**Methods:**

SF was collected from the knees of 45 OA, 22 RA patients and 20 asymptomatic organ donors. Eight biomarkers were selected and analyzed by using an enzyme-linked immunosorbent assay: interleukin (IL)-1, IL-6, IL-8 and IL-11; leukemia-inhibitory factor (LIF); cartilage oligomeric protein (COMP); osteocalcin; and osteogenic protein 1 (OP-1). Data are expressed as medians (interquartile ranges). The effects of sex and disease activity were assessed on the basis of the Western Ontario and McMaster Universities index score for patients with OA and on the basis of white blood cell count, erythrocyte sedimentation rate and C-reactive protein level for patients with RA.

**Results:**

The mean ages (± SD) of the patients were as follows: 53 ± 9 years for patients with OA, 54 ± 11 years for patients with RA and 52 ± 7 years for asymptomatic organ donors. No effect of participants' sex was identified. In the SF of patients with RA, four of five cytokines were higher than those in the SF of patients with OA and those of asymptomatic organ donors. The most significant differences were found for IL-6 and IL-8, where IL-6 concentration in SF of patients with RA was almost threefold higher than that in patients with OA and fourfold higher than that in asymptomatic donor controls: 354.7 pg/ml (1,851.6) vs. 119.4 pg/ml (193.2) vs. 86.97 pg/ml (82.0) (*P *< 0.05 and *P *< 0.05, respectively). IL-8 concentrations were higher in SF of patients with RA than that in patients with OA as well as that in asymptomatic donor controls: 583.6 pg/ml (1,086.4) vs. 429 pg/ml (87.3) vs. 451 pg/ml (170.1) (*P *< 0.05 and *P *< 0.05, respectively). No differences were found for IL-11 in the SF of patients with RA and that of patients with OA, while a 1.4-fold difference was detected in the SF of patients with OA and that of asymptomatic donor controls: 296.2 pg/ml (257.2) vs. 211.6 pg/ml (40.8) (*P *< 0.05). IL-1 concentrations were the highest in the SF of RA patients (9.26 pg/ml (11.1)); in the SF of asymptomatic donors, it was significantly higher than that in patients with OA (9.083 pg/ml (1.6) vs. 7.76 pg/ml (2.6); *P *< 0.05). Conversely, asymptomatic donor control samples had the highest LIF concentrations: 228.5 pg/ml (131.6) vs. 128.4 pg/ml (222.7) in the SF of patients with RA vs. 107.5 pg/ml (136.9) in the SF of patients with OA (*P *< 0.05). OP-1 concentrations were twofold higher in the SF of patients with RA than those in patients with OA and threefold higher than those in asymptomatic donor control samples (167.1 ng/ml (194.8) vs. 81.79 ng/ml (116.0) vs. 54.49 ng/ml (29.3), respectively; *P *< 0.05). The differences in COMP and osteocalcin were indistinguishable between the groups, as were the differences between active and inactive OA and RA.

**Conclusions:**

Activation of selected biomarkers corresponds to the mechanisms that drive each disease. IL-11, LIF and OP-1 may be viewed as a cluster of biomarkers significant for OA; while profiling of IL-1, IL-6, IL-8, LIF and OP-1 may be more significant in RA. Larger, better-defined patient cohorts are necessary to develop a biomarker algorithm for prognostic use.

## Introduction

Synovial fluid (SF) biomarker measurement has begun to provide useful clinical information. It is well understood that SF plays an important role in the lubrication and nutrition of the articular joint and in the metabolism of cartilage and other connective tissues within the joint. Biomarkers in SF can be categorized as anabolic or catabolic. Understanding the relationship between catabolic and anabolic markers and their changes during the onset of joint diseases will help to identify the key biomarkers of diagnostic and/or prognostic value. The focus of the current study was on proinflammatory mediators, catabolic cytokines (interleukin (IL)-1, IL-6, IL-8 and IL-11) and local anabolic markers of cartilage and bone metabolism (leukemia-inhibitory factor (LIF), cartilage oligomeric protein (COMP), osteocalcin and osteogenic protein 1 (OP-1), also called bone morphogenetic protein 7 (BMP-7)) that are involved in critical biological processes, including cell growth and activation, inflammation, immunity and differentiation. Several cytokines, such as IL-1, IL-6 and IL-8, have been found in SF of patients with rheumatoid arthritis (RA). The best understood is IL-1, which appears to be critical in the susceptibility to and progression of osteoarthritis (OA) and which has been shown to contribute to the induction of proinflammatory mediators (IL-6 and IL-8), proteolytic enzymes, nitric oxide, prostaglandins and other mediators and effectors of tissue inflammation and destruction [1-3]. IL-1 concentrations have been shown to be elevated in animal models of OA [4], while the efficacy of IL-1 inhibitors has been tested in OA patients [5]. In addition, a variety of other cytokines may be important in OA pathophysiology. For example, IL-6 has been associated with OA independent of patient age or weight [6]. In contrast, the potential of IL-8 and IL-11 as biomarkers for OA or RA has been studied less. IL-8 is produced excessively by fibroblasts, macrophages and neutrophils in pathological conditions [6], while IL-11 is one of the anti-inflammatory cytokines. An imbalance between proinflammatory and anti-inflammatory cytokines may result in the development of OA [7]. LIF is a glycoprotein that was originally defined by its ability to induce the terminal differentiation of murine M1 myeloid leukemia cells, resulting in the inhibition of their growth. LIF plays an important role in the induction of acute phase protein synthesis, in the regulation of both bone formation and bone resorption and in the degradation of proteoglycans. It has been detected at high concentrations in SF of patients with RA [8]. Another biomarker of interest is COMP, a member of the thrombospondin family of extracellular proteins, which is abundantly expressed in human cartilage. COMP has been extensively evaluated as a biomarker of joint tissue turnover in animals and humans, where its concentration in the SF or serum appears to reflect OA severity [9,10].

Prior studies have shown that OP-1 has unique anabolic and anticatabolic activity [11]. OP-1 has been detected in normal human SF as well as in SF of OA and RA patients [9]. Furthermore, in cartilage, there is a strong negative correlation between the concentrations of OP-1 and those of the IL-6 family of chemokines (IL-8, IL-11 and LIF) [12], findings which influenced the choice of biomarkers for evaluation in the current study.

We hypothesize that the activation of SF biomarkers in OA and RA might be dependent on the mechanism that drives each disease and that OA and RA might be characterized by a distinct panel of catabolic and anabolic markers of inflammation and cartilage matrix metabolism. Thus, the objective of our present study was to identify the concentrations of selected biomarkers in SF in samples taken from patients with RA or OA as well as from asymptomatic organ donors and to correlate these values with pathogenesis and disease activity. The novelty of this study is in the use of SF from asymptomatic human organ donors. The rationale for using selected markers was based on previous studies in human cartilage and the correlation analysis between markers of catabolism and anabolism for each disease.

## Materials and methods

### Study design

This study was approved by the institutional review board for human investigations at Rush University Medical Center. After securing informed consent from the participants, SF was obtained from 45 OA patients and 22 age-matched RA patients seen in the outpatient offices of the Rush University Section of Rheumatology who were undergoing diagnostic or therapeutic arthrocentesis as part of their evaluation and therapy. The patient cohort covered a broad spectrum of age and disease severity (both RA and OA), and all participants from many racial and ethnic backgrounds were recruited. Specific eligibility criteria are described below. SF samples were also obtained through the Gift of Hope Organ & Tissue Donor Network (Elmhurst, IL, USA) within 24 hours of death from 20 asymptomatic organ donors with no documented history of joint disease. The causes of death were cardiopulmonary arrest (*n *= 9), myocardial infarction (*n *= 5), liver failure (*n *= 1), gunshot wound (*n *= 1), suicide (*n *= 1), seizure (*n *= 1), intracranial bleeding (*n *= 1) and gastrointestinal bleeding (*n *= 1).

### Inclusion criteria for OA and RA participants

Inclusion criteria for recruitment into the study were age ≥21 years for all participants. Patients with RA had to fulfill the American College of Rheumatology (ACR) criteria for the diagnosis of RA [13]. Patients with OA had to fulfill the ACR criteria for the diagnosis of OA [14].

### Exclusion criteria

Patients with concurrent diagnoses of OA and RA and those with rheumatological disorders other than OA or RA that could influence their joint symptoms or inflammation were excluded to ensure better-defined experimental groups. Patients for whom arthrocentesis was not possible, or, when it was performed, did not yield a sufficient volume of SF were excluded. Patients who were unable or unwilling to provide informed consent for the study or for arthrocentesis and patients who were unable to read or understand the questionnaires were also excluded.

### Study protocol

Patients were screened for eligibility and provided their informed consent as described above. OA participants were evaluated on the basis of Western Ontario and McMaster Universities (WOMAC) index score to assess their symptoms and disease activity. Standard weight-bearing radiographs of the knees (standing anteroposterior and lateral views) were obtained from OA and RA patients. Radiographic OA was defined as the presence of Kellgren-Lawrence (K-L) grade ≥2 [15]. SF obtained from the arthrocentesis of the symptomatic knee was immediately transferred to our research laboratory and stored at -80°C. Participants' medical records, physical histories and laboratory data were reviewed by the study physician. Complete blood count, complete metabolic profile, C-reactive protein (CRP) level and erythrocyte sedimentation rate (ESR) were obtained for each participant. For RA subjects, rheumatoid factor (RF) was also obtained.

### Biomarker analysis

**Figure 1:**
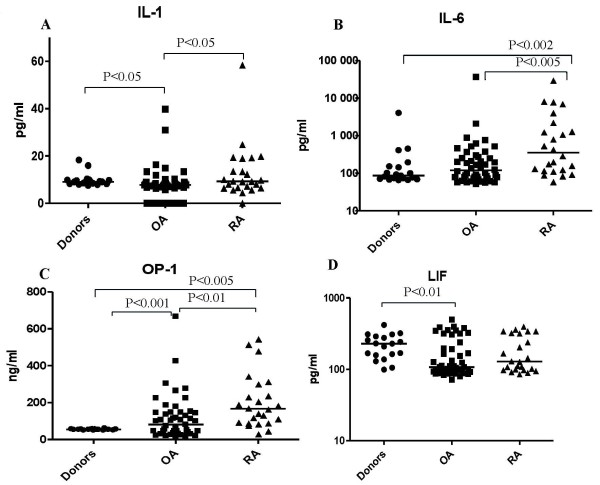
**ELISA data for IL-1, IL-6, OP-1, and LIF measured in SF collected from patients with OA or RA and from asymptomatic organ donors**. Quantitative ELISA data for **(A) **IL-1, **(B) **IL-6, **(C) **OP-1 and **(D) **LIF measured in synovial fluid collected from the patients with RA or OA or from asymptomatic organ donors. The data are presented as median scatterplots, where each point represents an average of three measurements.

SF from patients and organ donors was evaluated for IL-1, IL-6, IL-8, IL-11, LIF, COMP and osteocalcin by using commercially available enzyme-linked immunosorbent assay (ELISA) kits for each biomarker (R&D Systems, Inc., Minneapolis, MN, USA). The OP-1 assay was performed by using the ELISA method developed in our laboratory as previously described [16]. The data are presented in Figure 1 as median scatterplots, where each point represents an average of three measurements.

### Statistical analysis

All measurements were carried out in triplicate. All data were entered into a password-protected computer database. A χ^2 ^test or Student's *t*-test were performed for the OA, RA and asymptomatic organ donor groups to compare baseline demographics as well as clinical and laboratory data. Kruskal-Wallis and unpaired two tailed Mann Whitney tests were used to compare the concentrations of biomarkers in SF samples between the three experimental groups: OA vs. RA vs. donor. Data are presented as median (interquartile range) values. Spearman's correlation coefficient was used to correlate the concentrations of biomarkers against the appropriate laboratory parameters (for example, K-L grade, WOMAC index score, WBC count), and statistical significance was determined using the Bonferroni correction for multiple comparisons. Graphs were generated in Prism 3.0 software (GraphPad Software, La Jolla, CA, USA). *P *≤ 0.05 was considered statistically significant in two-tailed tests. More than 80% power was estimated to detect a 1.5-fold difference in various biomarker concentrations for the given sample size.

## Results

### Demographics of the sample population

SF from 45 patients diagnosed with OA (six males and thirty-nine females; mean age (± SD), 53 ± 9 years) and 22 RA patients (five males and seventeen females; mean age (± SD), 54 ± 11 years) were entered into the study. Twenty asymptomatic organ donors (five males and fifteen females; mean age (± SD), 52 ± 7 years) were also included. This study comprised age-matched population groups, and no sex differences were detected regarding the level of selected biomarkers within each cohort. The detailed demographics and baseline characteristics of the study participants are outlined in Table 1.

**Table 1:** Demographics and baseline characteristics of patients with OA or RA and of asymptomatic organ donors^a^

Patient demographics	OA (*n *= 45)	RA (*n *= 22)	Donors (*n *= 20)
Mean age, yr (±SD)	53 ± 9	54 ± 11	52 ± 7
Sex, M/F	6/39	5/17	5/15
WOMAC score	95 to 1,828	NA	NA
ESR, mm/hour	NA	10 elevated, 12 normal	NA
X-ray (K-L grade)	2-4	NA	NA
RF, IU	NA	All positive	NA
SF, WBC/μL	<2,000	7,000 to 25,000	<2,000

^a^OA, osteoarthritis; RA, rheumatoid arthritis; Donors, asymptomatic organ donor; ESR, erythrocyte sedimentation rate; WOMAC, Western Ontario and McMaster Universities (WOMAC) index; K-L grade, Kellgren-Lawrence grade; RF, rheumatoid factor; IU, international units; SF WBC, synovial fluid white blood cell count; NA, not applicable. The ages of patients and donors are mean age at study entry ± SD.

### SF IL-1 concentrations

The concentrations of IL-1 differed significantly between the RA and OA groups (9.26 (11.1) pg/ml vs. 7.76 (2.6) pg/ml; *P *< 0.05), and between the OA and asymptomatic organ donor groups (7.76 (2.6) pg/ml vs. 9.08 (1.6) pg/ml; *P *< 0.05) (values reported are medians (interquartile ranges)). The RA and asymptomatic organ donor groups were not significantly different (*P *= 0.93). In contrast to the IL-6, IL-8 and IL-11 concentrations, IL-1 concentrations were lower in the OA SF samples than in the asymptomatic donor samples (Figure 1A and Table 2).

**Table 2:** Synovial fluid concentrations of the biological markers in patients with OA or RA and in asymptomatic organ donors^a^

				*P *values		
				
Marker	Donor (*n *= 20)	OA (*n *= 45)	RA (*n *= 22)	OA vs. donor	RA vs. donor	OA vs. RA
IL-6, pg/mL	86.97 (82.0)	119.4 (193.2)	354.7 (1851.6)	0.4	0.0011	0.0026
IL-8, pg/mL	451 (170.7)	429 (87.3)	583.6 (1086.4)	0.0809	0.016	<0.0001
IL-11, pg/mL	211.6 (40.8)	296.2 (257.2)	217.5 (178)	0.0067	0.4557	0.2393
IL-1, pg/mL	9.08 (1.6)	7.76 (2.6)	9.26 (11.1)	0.02	0.9318	0.0497
OP-1, ng/mL	54.49 (29.3)	81.79 (116.0)	167.1 (194.8)	<0.0001	<0.0001	0.0013
LIF, pg/mL	228.5 (131.6)	107.5 (136.9)	128.4 (222.7)	0.0063	0.139	0.1991
Osteo, ng/mL	2.58 (6.6)	2.515 (4.9)	1.99 (3.9)	0.7437	0.893	0.5384
COMP, U/L	413.2 (339.9)	490.2 (207.5)	507.3 (352.9)	0.4652	0.3102	0.3865

^a^OA, osteoarthritis; RA, rheumatoid arthritis; IL, interleukin; OP-1, osteogenic protein 1; LIF, leukemia-inhibitory factor; Osteo, osteocalcin; COMP, cartilage oligomeric protein. Absolute values of biomarkers detected in synovial fluid of patients with osteoarthritis, rheumatoid arthritis and asymptomatic organ donors. *P *values were calculated by using an unpaired, two-tailed Mann-Whitney *t*-test with 95% confidence interval. Values are expressed as medians (interquartile ranges).

### SF IL-6 concentrations

SF IL-6 concentrations were significantly higher in RA patients than in OA patients or asymptomatic organ donors. In RA SF, IL-6 concentrations (354.7 (1,851.6) pg/ml) were threefold higher than in the OA samples (119.4 (193.2) pg/ml; *P *< 0.05), and they were fourfold higher than in the asymptomatic organ donor samples (86.97 (82.0) pg/ml; *P *< 0.05). Surprisingly, there was no statistically significant difference in the IL-6 concentrations between the OA and asymptomatic organ donor groups (Figure 1B and Table 2).

### SF IL-8 concentrations

SF concentrations of IL-8 were significantly higher in RA patients (583.6 (1,086.4) pg/ml) than in OA patients (429 (87.3) pg/ml; *P *< 0.05) and in the asymptomatic organ donor population (451 (170.7) pg/ml; *P *< 0.05). As with IL-6, there was no statistically significant difference in the IL-8 concentrations between the OA and asymptomatic organ donor samples (Table 2).

### SF IL-11 concentrations

No significant differences were found in the concentrations of IL-11 between RA and OA SF samples (217.5 (178) pg/ml vs. 296.2 (257.2) pg/ml; *P *= 0.239). However, OA IL-11 concentrations were higher than those in the asymptomatic organ donor samples (296.2 (257.2) pg/ml vs. 211.6 (40.8) pg/ml; *P *< 0.05); (Table 2).

### SF OP-1 concentrations

Concentrations of OP-1 measured in SF in this study were comparable to those previously described [9]. In the asymptomatic organ donor samples (54.49 (29.3) ng/ml), the concentrations of OP-1 were about threefold lower than those in the RA samples (167.1 (194.8) ng/ml; *P *< 0.05) and almost twofold lower than those in the OA samples (81.79 (116.0) ng/ml; *P *< 0.05) (Figure 1C). Moreover, as expected, the RA group had higher SF OP-1 concentrations than the OA group (twofold difference; *P *< 0.05) (Table 2).

### SF LIF concentrations

Contrary to other biomarkers measured in this study, the concentrations of LIF were significantly higher in the asymptomatic organ donor group (228.5 (131.6) pg/ml) than those detected in the OA group (107.5 (136.9) pg/ml; *P *< 0.05), though compared to the RA group, these concentrations were not significantly different (128.4 (222.7) pg/ml; *P *= 0.14). There was no significant difference between the RA and OA groups as well (*P *= 0.199) (Figure 1D and Table 2).

### SF osteocalcin concentrations

Osteocalcin concentrations were comparable in all three experimental groups: 2.58 (6.6) ng/ml for asymptomatic organ donor samples, 2.52 (4.9) ng/ml for the OA group and 1.99 (3.9) ng/ml for the RA group (Table 2).

### SF COMP concentrations

As with osteocalcin, concentrations of COMP were also indistinguishable between the three experimental groups: 413.2 (339.9) U/L in the asymptomatic organ donor group, 490.2 (207.5) U/L in the OA group and 507.3 (352.9) U/L in the RA group (Table 2).

### Correlation analysis of the biomarker concentrations identified in RA samples

Strong positive correlations were seen between IL-6 and IL-8 (Spearman's ρ = 0.86, *P *< 0.001), IL-1 and OP-1 (Spearman's ρ = 0.58, *P *= 0.003) and LIF and osteocalcin (Spearman's ρ = 0.59, *P *= 0.002) (Table 3). The SF WBC count positively correlated with IL-6, IL-8 and LIF (Spearman's ρ = 0.70, *P *< 0.001; Spearman's ρ = 0.57, *P *= 0.042; and Spearman's ρ = 0.52, *P *= 0.032, respectively) (Table 3). Only the IL-6 and IL-8 correlations remained statistically significant after applying the Bonferroni correction for multiple comparisons. No significant differences were observed in the concentrations of studied biomarkers between the patients with positive or negative RF.

**Table 3:** Spearman's rank correlation coefficients for RA study population^a^

	IL-1	IL-6	IL-8	IL-11	LIF	Osteocalcin	COMP	OP-1
IL-1	1.0000							
IL-6	ρ = 0.3643 *P *= 0.0801	1.0000						
IL-8	ρ = 0.5174 *P *= 0.0096	ρ = 0.8643 *P *= 0.001	1.0000					
IL-11	ρ = -0.2278 *P *= 0.2843	ρ = -0.1530 *P *= 0.4753	ρ = -0.0626 *P *= 0.7713	1.0000				
LIF	ρ = -0.1802 *P *= 0.3996	ρ = 0.2733 *P *= 0.1963	ρ = 0.3803 *P *= 0.0667	ρ = 0.2124 *P *= 0.3191	1.0000			
Osteocalcin	ρ = -0.0670 *P *= 0.7559	ρ = 0.1878 *P *= 0.3795	ρ = 0.1670 *P *= 0.4355	ρ = 0.3783 *P *= 0.0684	ρ = 0.5927 *P *= 0.0023	1.0000		
COMP	ρ = 0.3009 *P *= 0.1531	ρ = 0.0991 *P *= 0.6449	ρ = 0.0548 *P *= 0.7993	ρ = -0.2009 *P *= 0.3466	ρ = -0.3307 *P *= 0.1145	ρ = -0.0548 *P *= 0.7993	1.0000	
OP-1	ρ = 0.5791 *P *= 0.0030	ρ = 0.2896 *P *= 0.1699	ρ = 0.4130 *P *= 0.0448	ρ = -0.1896 *P *= 0.3750	ρ = 0.1897 *P *= 0.3746	ρ = 0.1817 *P *= 0.3954	ρ = 0.1922 *P *= 0.3683	1.000

^a^RA, rheumatoid arthritis; IL, interleukin; LIF, leukemia-inhibitory factor; COMP, cartilage oligomeric protein; OP-1, osteogenic protein 1. Spearman's rank correlation coefficients (IL-1, IL-6, IL-8, IL-11, LIF, osteocalcin, COMP and OP-1) for the RA cohort are expressed as Spearman's ρ and *P *values. A total of 22 RA patients were sampled, five male and seventeen female.

### Correlation analysis of the biomarker concentrations identified in OA samples

In contrast to a positive correlation between the IL-6 and IL-8 concentrations in RA patients (Table 3), in OA samples (Table 4) IL-6 and IL-8 correlated negatively (Spearman's ρ = -0.37, *P *= 0.001). IL-6 also strongly correlated with IL-11 and LIF (Spearman's ρ = 0.54, *P *< 0.001; and Spearman's ρ = 0.72, *P *< 0.001, respectively) and moderately correlated with osteocalcin (Spearman's ρ = 0.45, *P *= 0.001) (Table 4). There was also a strong negative correlation between the SF LIF concentrations and the IL-1 as well as IL-8 concentrations (IL-1: Spearman's ρ = -0.52, *P *= 0.0001; IL-8: Spearman's ρ = -0.04, *P *= 0.004) (Table 4). In contrast, LIF concentrations positively correlated with the concentrations of IL-6, IL-11 and osteocalcin (Spearman's ρ = 0.72, *P *< 0.001; Spearman's ρ = 0.43, *P *= 0.002; and Spearman's ρ = 0.50, *P *= 0.0003, respectively) (Table 4). IL-1 strongly correlated with IL-8 (Spearman's ρ = 0.62, *P *< 0.001) and showed moderate negative correlations with IL-6 and osteocalcin (Spearman's ρ = -0.33, *P *= 0.186; and Spearman's ρ = -0.034, *P *= 0.02) (Table 4). IL-8 and osteocalcin showed a moderate negative correlation (Spearman's ρ = -0.44, *P *= 0.001). COMP and OP-1 did not show any correlation with other biomarkers (Table 4). After the Bonferroni correction for multiple comparisons was applied, the correlations between IL-6 and IL-1, IL-6 and IL-8, osteocalcin and IL-1, osteocalcin and IL-11, and IL-8 and LIF lost the level of significance. A moderate positive correlation was detected between the SF WBC count and the IL-1 and IL-6 concentrations (Spearman's ρ = 0.4, *P *= 0.03; and Spearman's ρ = 0.37, *P *= 0.005, respectively), but no correlation was seen after the Bonferroni correction for multiple comparisons was applied. No correlations were identified between WBC count and IL-8, IL-11, LIF, OP-1, COMP or osteocalcin. No significant differences were detected in tested biomarkers in OA patients with active or inactive disease as defined by WOMAC index score, and no correlation was found between the biomarkers and the grade of radiological damage defined by the K-L score.

**Table 4:** Spearman rank correlation coefficients for OA study population^a^

	IL-1	IL-6	IL-8	IL-11	LIF	Osteocalcin	COMP	OP-1
IL-1	1.0000							
IL-6	ρ = -0.3386 *P *= 0.186	1.0000						
IL-8	ρ = 0.6233 *P *= 0.001	ρ = -0.3709 *P *= 0.0095	1.0000					
IL-11	ρ = -0.2312 *P *= 0.1136	ρ = 0.5384 *P *= 0.0001	ρ = -0.2365 *P *= 0.1056	1.0000				
LIF	ρ = -0.5241 *P *= 0.0001	ρ = 0.7165 *P *= 0.001	ρ = -0.4012 *P *= 0.0047	ρ = 0.4352 *P *= 0.0020	1.0000			
Osteocalcin	ρ = -0.3432 *P *= 0.0169	ρ = 0.4538 *P *= 0.0012	ρ = -0.4456 *P *= 0.0015	ρ = 0.3094 *P *= 0.0324	ρ = 0.5043 *P *= 0.0003	1.0000		
COMP	ρ = 0.0807 *P *= 0.5856	ρ = 0.0294 *P *= 0.8427	ρ = -0.1522 *P *= 0.3018	ρ = 0.1623 *P *= 0.2704	ρ = -0.0854 *P *= 0.5639	ρ = 0.1526 *P *= 0.3004	1.0000	
OP-1	ρ = 0.1038 *P *= 0.4828	ρ = 0.0730 *P *= 0.6222	ρ = -0.0529 *P *= 0.7211	ρ = 0.2028 *P *= 0.1669	ρ = 0.1003 *P *= 0.4975	ρ = 0.2084 *P *= 0.1552	ρ = 0.1193 *P *= 0.4193	1.000

^a ^OA, osteoarthritis; IL, interleukin; LIF, leukemia-inhibitory factor; COMP, cartilage oligomeric protein; OP-1, osteogenic protein 1. Spearman's rank correlation coefficients (IL-1, IL-6, IL-8, IL-11, LIF, osteocalcin, COMP and OP-1) for the OA cohort are expressed as Spearman's ρ and *P *values. A total of 45 OA patients were sampled, six male and thirty-nine female.

## Discussion

The usefulness of SF and/or serum-soluble biomarkers in the assessment of treatment efficacy or for monitoring disease progression in RA and OA remains controversial. The goal of the present study was to characterize and compare the concentrations of selected biological mediators (both catabolic and anabolic) in the SF obtained from RA or OA patients in comparison to SF aspirated from the knee joints of asymptomatic organ donors and to investigate whether any of these molecules might have utility as prognostic markers. Markers of catabolism included IL-1β and the IL-6 family of chemokines (IL-6, IL-8, IL-11 and LIF), which have been shown in cartilage to negatively correlate with OP-1 [12], the anabolic marker evaluated here. In addition, COMP and osteocalcin were included as markers of matrix metabolism. Our results suggest that the concentrations of the pathophysiologically important biomarkers in SF are different between OA and RA and depend on the mechanisms that drive cellular responses in each disease. IL-11, LIF and OP-1 appear to be significant for OA processes, while IL-1, IL-6, IL-8 and OP-1 may play an important role in RA.

In line with other studies [17,18], our data also indicate that RA, but not OA, is characterized by elevated IL-6 and IL-8 concentrations. The role of IL-6 in OA is unclear, and there are conflicting findings in the literature that indicate both procatabolic [19,20] and anticatabolic effects of IL-6 in chondrocytes and synoviocytes [21-23]. In contrast, the role of IL-6 in inflammatory processes, including RA, appears to be more consistent and involves promotion of the disease by stimulating B and T cells [24,25]. Furthermore, the concentrations of IL-6 in SF were shown to positively correlate with those in the sera of patients with RA, OA, crystal deposition and other forms of inflammatory arthritis [26]. As expected, IL-6 concentrations in biological fluids of asymptomatic organ donors were at the baseline concentrations and were significantly lower than those in patients with RA or OA. Similar to IL-6, IL-11 concentrations have been found to be significantly higher in SF than in serum, though the concentrations are highly correlated. In the present study, IL-11 concentrations were 1.4-fold higher in OA samples than those in asymptomatic organ donor samples, but not higher than those in the RA group. Trontzas *et al. *[27] reported that SF IL-11 concentrations are higher in OA than in treated RA, but not in untreated RA. As we did not distinguish treated from untreated RA, we were not able to confirm this relationship. Although elevated concentrations of IL-11 have been found in RA, limited data are available on this cytokine [27]. Still, our results suggest that further studies of the potential utility of IL-11 as a biomarker are warranted.

LIF, a cytokine in the IL-6 family, is downregulated by OP-1 and plays a role in bone formation and resorption. It has not been well-studied as a potential biomarker, and its association with OA has been based mainly on gene expression studies in synoviocytes [28]. In the present study, LIF concentrations were significantly lower in both the OA and RA groups compared to the organ donors. This finding differs from that in a previous report that detected elevated SF LIF concentrations in some patients with severe RA [29]. This discrepancy may be attributed to an inhibitory effect of other cytokines on LIF, for example, IL-4 [30]. Nonetheless, the difference in LIF response in OA relative to the other members of the IL-6 family (IL-8 and IL-11) suggests that LIF is either involved at different stages of the diseases or has a distinct function.

The cytokine that has received the most attention among the arthritic diseases is IL-1β, yet as a biomarker, it has been studied more in either experimental OA [31] or SF of patients with RA [32]. In OA, it is primarily viewed as a mediator of degenerative processes in human joint tissues [33-36], and substantial knowledge has been accumulated regarding its expression in cartilage and synovium, the mechanisms of its activation and interactions, its signaling, its regulation of and relationship with other active molecules, and so on. IL-1β, together with IL-6, has been shown in OA synovium to contribute to the progression of the disease by enhancing the susceptibility of chondrocytes to stimulation with proanabolic mediators [37]. In posttraumatic OA, especially in acute phase responses, IL-1β together with tumor necrosis factor α and IL-6 are well-established regulators of cartilage degradation and resorption [1]. As anticipated, in the current study, IL-1β and IL-6 concentrations were greater in RA patients than in OA patients or asymptomatic organ donors. However, to our surprise, the concentrations of IL-1β in SF of asymptomatic organ donors were statistically higher than those in OA, suggesting that IL-1β is involved only during the acute phase of the disease or is needed to initiate or trigger catabolic events. It is also a possibility that OA patients enrolled in our study underwent pharmacological treatment that had an inhibitory effect on IL-1β production or that only a subpopulation of patients with OA may have elevated IL-1β. The latter hypothesis is supported by a recent publication by Neu *et al. *[38], who reported elevated concentrations of IL-1β in only a few OA samples, while in other samples IL-1β either was barely detectable or was below the detection limit. Though in previous publications IL-1β and IL-6 have been shown to be predictive of either OA or RA, our data indicate a closer association of both cytokines with RA than with OA.

As markers of matrix metabolism, we used COMP and osteocalcin. COMP is an extracellular glycoprotein and is a member of the thrombospondin family of calcium-binding proteins. COMP is associated with cartilage breakdown and has been studied as a potential diagnostic and prognostic indicator as well as a marker of disease severity or the efficacy of treatment (reviewed in [39]). It has been reported that COMP concentrations in SF are 10 times higher than in serum and that higher COMP concentrations have been observed in patients with higher radiographic Kellgren-Lawrence grades. However, despite these expectations, here we were not able to identify an association of COMP concentrations with the type of disease or its severity, perhaps because of the limitations of our study. Samples were collected at only one time point rather than longitudinally, there was a lack of untreated controls and/or the sample size of each group was not large enough. As with COMP, we did not find differences in osteocalcin concentrations between the experimental groups, though elevation of osteocalcin has been detected previously in the destructive form of OA in comparison with nondestructive OA [40]. In agreement with our data, the observations of Salisbury *et al. *[41] suggested that in a predominantly older female population, the rate of normal bone turnover measured by osteocalcin in donors was not significantly different from that of OA or RA patients. Furthermore, it has been reported that OA and RA patients treated with nonsteroidal anti-inflammatory drugs showed significantly lower concentrations of SF osteocalcin than patients treated with glucocorticoids [42]. Conflicting data on both COMP and osteocalcin indicate that only carefully designed longitudinal studies with well-controlled, large patient cohorts may shed the light on their potential as biomarkers.

Previously, we described in detail OP-1/BMP-7 in SF from organ donors or OA and RA patients [9]. In this study, it was used primarily for correlation with other catabolic markers or because there are fewer anabolic than catabolic biomarkers, where OP-1/BMP-7 definitely belongs to the former category. As earlier, we confirmed the elevated concentrations of OP-1/BMP-7 released into the SF of RA patients in comparison to OA patients and organ donors. A higher quantity of OP-1/BMP-7 in samples characterized by higher concentrations of proinflammatory mediators may not necessarily indicate a higher synthesis of this growth factor. Our unpublished data suggest that treatment with IL-1β, for example, induce activation of pro-OP-1 and thus release of active OP-1 from the matrix. In addition, catabolic mediators lead to matrix loosening/degradation, which also may favor activation and/or release of the growth factor that has been trapped within the matrix or bound to the extracellular binding proteins or matrix components as it occurs with the transforming growth factor β latency protein [43].

Biomarkers were also assessed on the basis of the activity of the disease within each patient cohort. OA was assessed on the basis of the WOMAC index score, and RA was evaluated on the basis of the synovial WBC count, ESR and CRP level. Though no significant differences between biomarkers and disease activity were found, there was a trend toward an elevation of proinflammatory mediators in the active state of OA or RA.

## Conclusions

In conclusion, the results of this study point to the mechanism-specific activation of biomarkers, where RA associated with higher inflammatory components is characterized by a profile of elevated IL-1, IL-6, IL-8 and OP-1 as well as low concentrations of LIF. On the basis of our studies, the OA profile of biomarkers could be described as elevated concentrations of IL-11 and OP-1 and low concentrations of LIF. To move forward in the field of biomarkers, the criteria for study design should be more stringent and should include larger, well-defined patient cohorts, preferably without any accompanying therapeutic interventions that obscure the accuracy of analysis.

## Abbreviations

ACR: American College of Rheumatology; BMP-7: bone morphogenetic protein 7; CBC: complete blood count; CMP: complete metabolic profile; COMP: cartilage oligomeric protein; CRP: C-reactive protein; ELISA: enzyme-linked immunosorbent assay. ESR: erythrocyte sedimentation rate; IL: interleukin; K-L: Kellgren-Lawrence; LIF: leukemia-inhibitory factor; OA: osteoarthritis; OP-1: osteogenic protein 1; RA: rheumatoid arthritis; RF: rheumatoid factor; SF: synovial fluid; WBC: white blood cell count; WOMAC: Western Ontario and McMaster Universities.

## Competing interests

Stryker Biotech provided research support for studying the biomarkers in the synovial fluid of patients with RA and OA as well as that of asymptomatic organ donors.

## Authors' contributions

RK, a fellow in the Section of Rheumatology, Department of Internal Medicine, was responsible for the recruitment of patients, evaluation of their medical histories, performing arthrocentesis and drafting the manuscript. RA, a fellow in the Section of Rheumatology, Department of Internal Medicine, performed the correlation and statistical analyses of the data and was involved in drafting the manuscript. SL, a postdoctoral fellow in the Department of Biochemistry, was involved in data acquisition and organization as well as manuscript preparation. AH, the research assistant/laboratory manager at the Department of Biochemistry, was responsible for the handling and preparation of samples and ELISA analysis. DR, a senior director of research and development at Stryker Biotech, was involved in the conceptual development of the project as well as its objectives, specific aims and experimental design. JB, director of the Section of Rheumatology and the fellowship program, provided mentorship support to the fellows, was involved in the development of the project and its acquisition as well as in the preparation of the manuscript. SC, the principal investigator of the project, developed the study's conceptual idea, wrote the research proposal, obtained research funding and institutional review board approval, oversaw the progress of the study and acquisition of the project-related data, coordinated the efforts of the study participants, wrote progress reports sent to the funding agency (Stryker Biotech and Ciba-Geigy Endowed Chair), and was involved in the final editing of the manuscript.
